# Homeostasis as a proportional–integral control system

**DOI:** 10.1038/s41746-020-0283-x

**Published:** 2020-05-22

**Authors:** Lennaert van Veen, Jacob Morra, Adam Palanica, Yan Fossat

**Affiliations:** 1 Faculty of Science, Ontario Tech University, Oshawa, ON Canada; 2 Labs Department, Klick Health, Klick Inc, Toronto, ON Canada

**Keywords:** Medical research, Biomarkers

## Abstract

According to medical guidelines, the distinction between “healthy” and “unhealthy” patients is commonly based on single, discrete values taken at an isolated point in time (e.g., blood pressure or core temperature). Perhaps a more robust and insightful diagnosis can be obtained by studying the functional interdependence of such indicators and the homeostasis that controls them. This requires quasi-continuous measurements and a procedure to map the data onto a parsimonious control model with a degree of universality. The current research illustrates this approach using glucose homeostasis as a target. Data were obtained from 41 healthy subjects wearing over-the-counter glucose monitors, and projected onto a simple proportional–integral (PI) controller, widely used in engineering applications. The indicators quantifying the control function are clustered for the great majority of subjects, while a few outliers exhibit less responsive homeostasis. Practical implications for healthcare and education are further discussed.

## Introduction

How does one measure and assess health? “Measurement” may be referred to as quantifying attributes or characteristics belonging to a patient, whereas “assessment” may be referred to as drawing qualitative conclusions from the data that were measured^1^. Traditionally, guidelines in the field of medicine have defined the traits that contribute to health as single, discrete values, or set ranges, often taken at a single time point^2^. This is true for many physiological functions, such as glycemia, core temperature, body mass index, bone density, cholesterol, and blood pressure. Although physicians may take into account various other factors of individual patients, the predetermined guidelines for medical diagnoses are confined to scoring heuristics. These values are measured and assessed using simple scoring gradients where any patient whose value falls into a particular range may be defined as “healthy”, and all others defined as “unhealthy”.

Although using simple heuristics to measure and assess health may be efficient and unambiguous, this approach does not explain the fundamental control mechanisms and physiological systems that lead to healthy values. Single values only measure the “what” of health and miss the “how”. For example, a blood pressure of 120/80 mm Hg may indicate a “healthy” value, but it is only taken at one time point in the patient’s day. This value gives no indication of how effective the body is at controlling blood pressure when handling physical or mental stress. In other words, the discrete, single time point values of physiological biometrics are merely manifestations of a deeper, more complex health control system.

Health may be better measured and assessed by studying the body’s ability to maintain homeostasis, i.e., the maintenance of specific variables within an optimal range, regardless of external stimuli^3^. Many of today’s most prevalent chronic illnesses, such as hypertension, diabetes, obesity, and depression, can be considered failures of the body’s ability to maintain homeostasis or keep physiological signals within a normal working range. Therefore, knowing the functional model of healthy homeostasis may yield a better understanding of the overall well-being of the patient, and could become a fundamental step toward a more refined assessment of health and an early warning signal for illness.

To advance this idea, it is critical to examine a patient’s individual homeostatic function and compare it to a base reference model to identify quantitative and qualitative deviations from healthy functioning. However, two challenges currently render this approach impractical:

- existing models of homeostatic systems are complex;
- the assessment of an individual’s homeostasis function lacks a simple scoring system.

As a solution, we propose to describe the homeostatic function as a *control system* wholly independent of the underlying physiology. Control systems are widely used in engineering, economics, and cybernetics, and provide a strategy for maintaining the state of a system within a safe range without reference to its detailed mechanics. In particular, we consider a control strategy based on the system’s current state and on its recent history. A scoring system can then be derived from the relative influence of these two factors for a given patient.

One process that can be considered a control system is that of glucose homeostasis. A dysfunction of glucose homeostasis is associated with diabetes. It is estimated that more than 422 million people worldwide have diabetes^4^, while more than 352 million others have prediabetes^5^. Diabetes is also associated with more than US$ 827 billion direct medical costs to the world every year^4^. Thus, an evaluation method to understand a patient’s glycemic homeostatic function may be a key first step in reducing the economic and social burden of diabetes.

The standard methodology of measuring glycemic dysfunction includes HbA1c measurements, fasting blood glucose tests, and oral glucose tolerance tests^6^. All three tests use simple heuristics to distinguish healthy patients from those with prediabetes or diabetes. Perhaps a better evaluation method to understand the nuanced structure of the glycemic system may be obtained by modeling its dynamic function. Although models of normal glycemic control currently exist, they tend to be fairly complicated. These models use a large number of variables and parameters, and describe a multitude of biophysical processes, rather than the resulting control strategy itself. For instance, the model recently proposed by Masroor et al.^7^ comprises 5 dynamical equations and over 25 parameters. The use of such models is limited by the “curse of dimensionality”, i.e., the catastrophic growth of the number combinations of parameter values to explore when attempting to reproduce measured data. We demonstrate that a simple model based on a feedback control can robustly reproduce suitably pre-processed data. From the model parameters resulting from the fitting procedure, we extracted dimensionless indicators that quantify the homeostatic control function.

The procedure of fitting the model parameters relies on the availability of a quasi-continuous stream of data over a period much greater than the time scales typical for glucose production and consumption. Recently, off-the-shelf continuous glucose monitoring (CGM) technology has become available to provide a convenient and cost-effective way to accurately measure continuous glycemia from subjects pursuing their regular daily activities. The present study utilized the FreeStyle Libre glucose monitor (Abbott Diabetes Care) on participants not diagnosed with any medical condition to gather glucose level data every 15 minutes for 2 weeks. From these data, we extracted a sequence of glucose levels representative of the subject’s feedback control, and then iteratively optimized the model parameters to reproduce it.

The objectives of the current research were threefold:

- to extract a personalized, functional description of a subject’s glucose homeostasis from easily obtained, quasi-continuous measurements;
- to confirm the universality across subjects of the homeostatic feedback system model;
- to extract medically actionable indicators from this functional description.

Overall, this research was intended to identify an objective indicator of glucose homeostasis that can be derived from time series data with no requirement to model the physiological details of the underlying system. The final algorithm was not intended to offer any control of glucose, but rather as a descriptive tool used to potentially assess the homeostasis health of participants. The ultimate goal of this research was to provide a different form of health assessment that could be used to identify potential risk factors in glucose tolerance impairment.

## Results

### A simple control model

Feedback control is a strategy to minimize the deviation of a process variable, in our case the glucose level, from a set value. A simple strategy, refered to as proportional–integral (PI) control, is based on a response proportional to this deviation and on an integral over its history. The application of PI control goes back at least as far as 1922, when N. Minorsky proposed to use it for the automatic steering of ships. Since then, it has been used to control processes as diverse as the pasteurization of milk at a constant temperature, and the balancing of flying drones^8,9^. We conjecture that the PI controller can effectively describe the homeostatic control system resulting from various physiological pathways independently from the details of any of these pathways. Our assumptions are the following:

- There is an instantaneous response in proportion to the deviation of the glucose level from the set point, for instance through the release of insulin or glucagon into the blood stream.
- There is memory in the system due to the finite time it takes the body to excrete or metabolize the hormones involved. It is reasonable to assume that the rate at which this happens is proportional to the hormone concentration, so that the memory fades exponentially in time.

The PI controller is coupled to a rudimentary model of blood glucose kinetics. It comprises only the effects of the base metabolic rate, food intake, and the control feedback. The feedback term takes the form of *mass action kinetics*. That is, it corresponds to the rate of a hypothetical chemical reaction between two substances with concentrations *u*, the control rate, and (*e* + *e*_sp_), the total glucose concentration, in a well-mixed reaction vessel under the assumption that the reaction takes place with constant probability every time different molecules collide. This form of the control term coincides with that proposed by Bergman et al.^10^, who tested several models of insulin-glucose interactions. In that study, a measured quantity of insulin was injected directly into the blood stream, and the insulin and glucose concentrations were measured at regular intervals afterward. The interaction term proportional to both the insulin and the glucose concentration was shown to best model the study.

If *e* denotes the deviation from the set point glucose concentration, *e*_sp_, the equations are

1 $$u={A}_{1}e+{A}_{2}{\int \nolimits_{t^{\prime} =-\infty }^{\infty }}w(t-t^{\prime} ,\lambda )e(t^{\prime} )\ {\rm{d}}t^{\prime} ,$$

2 $$\frac{{\text{d}}e}{{\text{d}}t}=-{A}_{3}+F(t)-u\ (e+{e}_{{\rm{sp}}}),$$

3 $$w(\tau ,\lambda ) = \left\{ {\begin{array}{*{20}{l}}0&{{\rm{if}}\,\,\tau \, < \,0}\\{\lambda \,{\rm{exp}}( - \lambda \tau )}&{{\rm{if}}\,\,\tau \, > \,0}\end{array}} \right.$$

Here, *F*(*t*) models the release of glucose into the blood stream and *w* models the sensitivity of the control variable to past glucose concentrations. We measure *e* and *e*_sp_ in units of mmol/L, while *u* has units of 1/Δ, where Δ = 15 mins is the interval of measurements taken by the monitoring system described in the Apparatus section. We assigned a constant value of ${A}_{3}=0.005\ {\rm{mmol}}/(\Delta \times {\rm{liter}})$, which corresponds to a base metabolic rate of about 1300–1950 kcal/24 hours and a blood volume of about 4.5–6.4 L. The parameters of the model are summarized in Table 1.

**Table 1:** Parameters of the glucose homeostasis model with their definition, typical range across test subjects, mean, and standard deviation across subjects after the fitting procedure described in the Results.

Parameter	Definition	Range	Mean	Standard Deviation	Units
*A*_1_	Proportional control term	−0.1–0.7	0.19	0.18	liter/(Δ × mmol)
*A*_2_	Integral control term	0.09–0.75	0.34	0.24	liter/(Δ × mmol)
*A*_3_	Basic metabolic rate	N/A	0.005	N/A	mmol/(Δ × liter)
*λ*	Decay rate of the Integral term	0.1–0.7	0.42	0.13	1/Δ

Here, Δ = 15 minutes is the interval between two measurements of the FreeStyle Libre flash glucose monitor.

Our model has some properties in common with that of Steil et al.^11^, who considered a proportional–integral–derivative (PID) control as a model for the secretion of insulin by β-cells. Like in our case, the controller is coupled to a simple model of glucose dynamics based on mass action kinetics. However, the coupling is not direct, but is effected through a first-order differential equation for the blood insulin concentration that, in turn, drives another first-order equation for the rate of glucose clearance. In contrast to our model, their control variable has a clear interpretation as the rate of insulin secretion. As a disadvantage, their model has two more variables and two more time scales as compared to ours. In a relatively small study (*n* = 7), they estimated the model parameters from data taken during a hyperglycemic clamp experiment. Validation of the PID-based approach under more realistic circumstances fell outside the scope of their work, and it was the topic of our current study.

### Reproducing measured data with the model

**Fig. 1:**
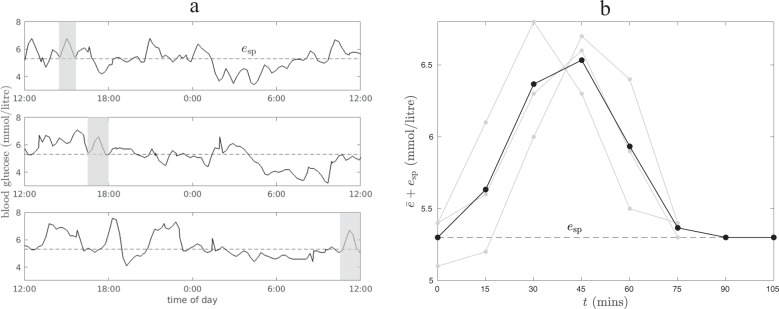
Construction of the representative peak for one subject. **a** Three days of raw data. The shaded time segments were averaged to find a representative peak. The dashed line corresponds to the set point glucose level *e*_sp_. **b** The three selected peaks (gray) and the representative peak (black). The set point is taken to be the minimum over the representative peak. Two data points at the set point are added to the representative peak at the end. The fit for this representative peak is shown in the rightmost inlay in Fig. 2.

For each subject, the parameter values of the PI model’s equations (1–3) are chosen to minimize the difference between the model output and measured glucose data. We did not fit the measured raw data as it may be variable due to noise. Instead, we selected a number of peaks of glucose and used their average as the representative peak for the subject. Averaging over too few data segments yields a representative peak with too much noise, while averaging over too many obfuscates the structure of the data. We found that three to five sample peaks were sufficient. In selecting the data segments by visual inspection, we found no pronounced dependence on the time of day at which the glucose excursion was triggered. While insulin sensitivity is known to have a circadian rhythm, this did not seem to affect our functional description of glucose homeostasis over the time scales considered here. Figure 1 shows raw data for one test subject. Three peaks in glucose, indicated by shading, have been selected to form the representative peak, $\bar{e}$, shown on the right of Fig. 1; the set point glucose level, *e*_sp_, corresponds to the minimum over the representative peak and is indicated by a dashed line.

Once the representative peak has been distilled from the raw data of a given subject, we tune the model parameters to reproduce it as accurately as possible. The accuracy of a fit is measured by the function

5 $$E=\frac{\mathop{\sum }\nolimits_{i = 1}^{{n}_{{\rm{peak}}}}{\left(\bar{e}({t}_{i})-e({t}_{i})\right)}^{2}}{\mathop{\sum }\nolimits_{i = 1}^{{n}_{{\rm{peak}}}}\bar{e}{\left({t}_{i}\right)}^{2}},$$

where *n*_peak_ is the number of points in the representative peak, Δ = 15 (mins) apart. This least-squares fit is computed by a steepest descent algorithm, the details of which are explained in the Supplementary Methods.

### Main findings

**Fig. 2:**
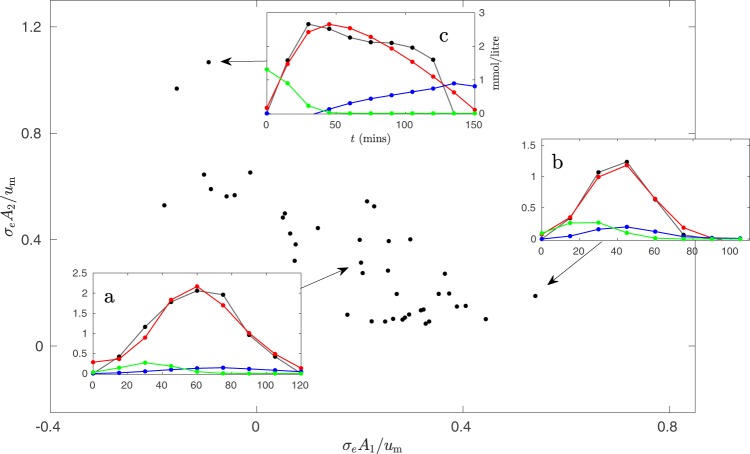
Scatter plot of the optimal model parameters for all subjects. Shown are *A*_1_ and *A*_2_, nondimensionalized by the standard deviation of each subject's time series of the glucose level, *σ*_*e*_, and the corresponding maximum of the control variable, *u*_m_. Three illustrative results of the fitting procedure are shown in inlays. In these, the black lines correspond to the representative peaks and the red lines to the output *e*(*t*) of the control model (1–3). The normalized input function, *F*(*t*)/*λ*, and the normalized control variable, *σ*_*e*_*u*(*t*)/*λ*, are shown in green and blue, respectively.

We computed the best fit for each of the 41 subjects, reaching a residual mismatch of *E* < 0.06 for all; for 90% of the subjects, the mismatch was less than 0.02. A scatter plot of the results is shown in Fig. 2. On the axes are the dimensionless parameters *σ*_e_*A*_1_/*u*_m_ and *σ*_e_*A*_2_/*u*_m_, where *σ*_e_ is the standard deviation of all glucose measurements for a given subject and *u*_m_ is the maximum attained by the control variable in the optimal fit. The mean values of *A*_1_ and *A*_2_ were 0.19 and 0.34, respectively, and their standard deviations were 0.18 and 0.24, respectively. The data for most participants were within one standard deviation from the mean for *A*_1_ (68%) and *A*_2_ (76%). There were two notable outliers, specifically for *A*_2_, in the top-left corner of Fig. 2, with parameters greater than 2.5 standard deviations away from the mean; no outliers were found for *A*_1_. Three inlays have been included to illustrate the qualitative difference between the fitted curves. Inlay a shows the data point close to the mean value for both parameters. The width of the model output peak *e*(*t*) was about 50 min and the control variable has a smooth peak, delayed by about 15 (mins), and decays back to zero about 1 hour after the glucose peak. If we take the control variable *u* to be a proxy for the insulin concentration, these numbers agree well with data for healthy subjects undergoing a meal glucose tolerance test presented by Caumo et al.^12^.

Inlay b shows a faster response to approximately the same input. While the amplitude of the input function is close to that in inlay a, the peak glucose level is almost two times lower. The width of this peak is only about 30 min and the control variable assumes its resting value within 30 min from the glucose peak value.

On the other end of the scale, Inlay c shows a relatively slow response. The measured data exhibit a plateau at a level of over 2 mmol/L in excess of the set point glucose level. While the model accurately captures the rapid rise over the first 30 min, the measured and modeled data diverge somewhat over this plateau. The control variable reaches a level that is about four times higher than that in inlays a and b and remains high after the measured and modeled glucose concentrations return to the set point.

These qualitative differences can be understood from the structure of the model. If *A*_1_, *A*_2_ > 0 and *A*_1_ > *A*_2_, the controller (1) is mostly determined by an instantaneous, proportional response. For a rapidly fluctuating glucose level, this may lead to fluctuations of the control feedback that cannot be sustained by any physiological mechanism, but for a peak generated by a regular meal, it leads to a quick reset. On the other side of the diagram, if *A*_1_ < 0 and *A*_2_ > 0, the integral and proportional terms of the control model have an opposite effect. This is the only way for our model to produce a sustained excess glucose level. In the first phase, when the representative peak is rapidly rising, the proportional term is dominant and gives rise to a positive feedback. This increases the model glucose level rapidly. In the second phase, the integral term increases until it balances the proportional term. For a period in the order of 1/*λ*, the glucose level remains elevated. Finally, the integral term becomes dominant and induces the system back down to the target level.

**Fig. 3:**
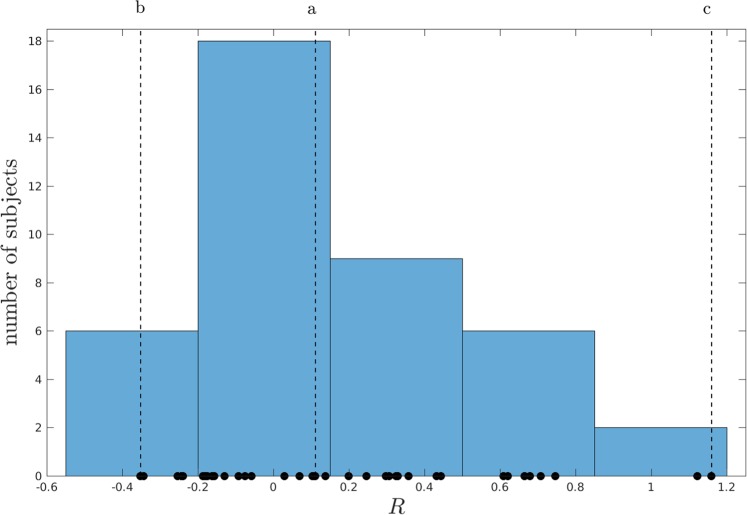
Distribution of the indicator *R* = *σ*_e_(*A*_2_ − *A*_1_)/*u*_m_ over test subjects. The number of subjects per bin is on the vertical axis, while individual data are indicated with dots on the horixontal axis. The dashed lines labeled a, b, and c correspond to the inlays in Fig. 2.

There was a strong negative correlation between the two model parameters (*A*_1_ and *A*_2_) in Fig. 2 (Pearson correlation, *r* = −0.81, *p* < 0.001). With this observation in mind, we devised a single indicator, *R* = *σ*_*e*_(*A*_2_ − *A*_1_)/*u*_m_, which indicates the responsiveness of the glycemic control systems. The *R* value of all subjects is shown in Fig. 3, with the values displayed as dots on the horizontal axis, and the distribution displayed as a histogram. Although more data are needed to establish the shape of this distribution, it appears to have a clear modal value of around *R* = 0, and a positive skew towards higher values. We speculate that this indicator may be used as an actionable diagnostic tool, extracted from quasi-continuous glucose measurements in real-time. However, future research would need to examine this question in more detail.

We also analyzed the correlations of the *A*_1_ and *A*_2_ variables to all demographic factors and found no significant associations with age, gender, or BMI across individuals (all *p* > 0.28). Thus, we can infer that our model is not greatly influenced by other variables within the participants.

## Discussion

The goal of this research was to propose an alternative technique of health measurement and assessment by examining homeostatic control systems rather than discrete, single values. For example, the gold standard methodologies of screening for type 2 diabetes or prediabetes include glycated hemoglobin (HbA1c) measurements, fasting plasma glucose (FPG), and plasma glucose during an oral glucose tolerance test (OGTT). All of these tests are evaluated against predetermined guidelines set by the medical community^6^. However, these different tests may reveal discrepant results across different patients of different backgrounds, and may be unreliable when trying to evaluate how glucose changes over time. Thus, our intent for this research was to provide a more insightful way of assessing and evaluating glucose levels by showing a larger window of data for the individual, and demonstrating the rate of change for those data, rather than a single point in time.

We also wanted to improve existing models of homeostatic systems by developing uncomplicated algorithms for extracting medically relevant insights and actions. In this case, our aim was to formulate a model of glucose homeostasis that emphasizes simplicity and universality over physiological detail. “Simple” refers to the fact that our model has only two variables (the control variable *u* and the excess glucose concentration *e*) and five parameters (those that regulate the control strategy, *A*_1_, *A*_2_, and *λ*, as well as the amplitude and shift of the input function). “Universal” refers to the fact that the model can be applied to all test subjects under differing circumstances, while no explicit reference is made to the pathways of glucose control, or potentially to other forms of homeostasis, like blood pressure or core temperature.

While a significant body of research exists in modeling glucose homeostasis^13^, the mathematical models that currently exist for humans or animals include a multitude of variables and physiological parameters. In the case of machine learning-based models, the result is individualized to each subject^14^. These features limit their use as a diagnostic tool in clinical settings where simplicity, interpretability, and universality are paramount.

Other research in this field has examined the closed-loop artificial pancreas and insulin pumps^15–17^. The main purpose of these digital therapeutic devices has been to treat patients with type 1 diabetes with a device that injects insulin or glucagon subcutaneously based on continuous measurement of blood glucose. Our research compliments and extends this previous work by examining a PI control model in healthy individuals. The great majority of research on homeostasis modeling has only examined patients who have been diagnosed with a known medical condition; virtually no research has been conducted on healthy controls to obtain a more insightful metric of health for enabling future prognosis of potential disease states. We believe that our research helps support past work, and could be applied to a greater proportion of the population for routine health assessments in normal, non-diagnosed individuals.

Additionally, in type 1 diabetes management, feedback control has been used primarily as a replacement of the glucose controller (i.e., pancreas), rather than a diagnosing tool based on a functional description of the glycemic system^18^^,^^19^. With an artificial pancreas, there is usually a significant delay (15–30 min) in the uptake of the glucagon and insulin after injection^18^. This leads to different models and different dynamics, precluding a direct comparison between closed-loop control models for an artificial pancreas and a healthy homeostatic function. Nevertheless, the successful application of PI or PID—including the rate of change of blood glucose—in the clinical setting attests to the universality which we advocate.

The current research demonstrates that the technique for processing glucose data from a portable device has low computational complexity, and can, in principle, be done in real-time. For example, this process would consist of a few straightforward steps:

- From the raw data, 3–5 peaks are selected of comparable width. The average of these is the representative peak for the subject in question.
- The model parameters are iteratively tuned to make the model output as similar as possible to the representative peak.
- From the optimized parameters and the output control variable, a dimensionless indicator is extracted that encodes the responsiveness of the control system.

The PI control strategy can provide a sophisticated model for examining glucose homeostasis in humans. The model parameters that are extracted give more detailed information than single, discrete values, like HbA1c, since they present information about the way a subject’s glucose level is being controlled rather than statistics on the glucose level itself.

It is worth noting that the amount of HbA1c may be described as a form of “memory”, since it provides an average blood glucose concentration estimate over the preceding 2 to 3 months. However, the test itself is only taken at one time point, and the overall value of HbA1c yields a singular, discrete number (i.e., average), with no indication of standard deviation or rate of change over the past few months. In other words, this test does not provide any graph or visualization of how the HbA1c level has gone up or down over time in the individual.

On the other hand, mathematical modeling unveils a form of universality that is found across applied sciences. In biophysics, for instance, “synchronization” can be found in a variety of systems, from firing neurons^20^ to signaling fireflies^21^. Similarly, pattern formation near critical transitions occurs in the same way across a range of settings, from electrical signals in cardiac tissue to density variations in bacterial populations^22^. Thus, models based on PI control could yield a parsimonious description of a variety of homeostatic functions, regardless of their particular mechanics.

We demonstrated that the extracted model parameters fall within a well-defined range. More precisely, for both *A*_1_ and *A*_2_ parameters, 68% and 76% of the subjects fall within one standard deviation of these means, respectively. Only two subjects had a parameter value that deviated more than 2.5 standard deviations from the *A*_2_ mean, while no outliers were found for *A*_1_. For the indicator *R*, the two outliers show representative peaks that show a relatively slow decay of glucose levels. For these outliers, the proportional and integral terms of the control strategy work against each other. We may speculate whether this indicates a pathological state such as prediabetes, but future research is needed to investigate this possibility; the current research utilized participants who were not diagnosed with any medical condition. Nevertheless, if it is the case that this PI control model does predict or diagnose some type of medical issue, it would give extra credibility to its usefulness in everyday practice.

Since this pilot study was exploratory in nature, there are some limitations which should be addressed with further research. Firstly, since the current data were collected from individuals not diagnosed with any medical condition, the indicators that rate the effectiveness of the controller may need to be refined in a sample of patients diagnosed with prediabetes or type 2 diabetes for comparison. Secondly, we have assumed that the input peak *F*(*t*) always takes a Gaussian form, since this assumption agrees reasonably well with data measured in vitro^23^. However, in order to turn our data processing pipeline into a diagnostic tool, we may need to allow for a wider class of input functions; more a priori knowledge of the food intake, for instance, would also help to model the release of glucose into the blood stream over time. Lastly, this study used the FreeStyle Libre glucose monitor due to convenience, as this study was performed in Canada, where the device is available without any prescription; the FreeStyle Libre measures glucose in the interstitial fluid (ISF), but not directly in the blood. While the glucose levels in the ISF are closely inline with blood glucose, a delay of 5–10 minutes has been estimated by various studies^24^. Nevertheless, this study shows that an off-the-shelf consumer level device may be used to redefine homeostasis measurement and assessment.

It should also be noted that although continuous glucose monitors provide real-time data and graphics, they do not provide a universal algorithm which can be scored and evaluated by physicians. The devices merely provide raw data, which the clinician must visually inspect subjectively to analyze any risk factors; it does not provide an objective indicator of glucose tolerance in a single snapshot, but rather across a larger window of time through multiple peaks and valleys. Our research was meant to take into account these multiple peaks and valleys to provide an overview of glucose homeostasis for each person.

This study contributes to the field of digital medicine by providing an improved method of understanding health beyond single values, and furthering our understanding of homeostasis in the normal population. Importantly, these techniques can be utilized in other patient populations and disease states. We believe that a personalized health measurement such as this one could lead to more patient education, engagement, and empowerment into their own personal health. Being able to better visualize one’s own physiological mechanisms could be the next step to better medical standards. These findings have practical implications for healthcare and education where enhanced translation of medical knowledge can empower both the physician and patient.

## Methods

### Participants

Data were collected from 41 participants (20 females; 21 males; age range = 19–50 years, M age = 32.4 years, SD = 6.8 years, see Supplementary Table 1). Participant race included 23 (56.1%) Caucasian, 15 (36.6%) Asian, 1 (2.4%) African American, 1 (2.4%) Hispanic, and 1 (2.4%) mixed race (Caucasian and African American). Exclusion criteria were participants below the age of 18, those who were diagnosed with any mental or physical medical condition of any kind (chronic or acute), those taking any form of prescription medication, and those who were pregnant or breastfeeding. This sample of participants had an average body mass index of 25.8 (SD = 5.7), an average resting blood pressure of 120/75 mm Hg, and an average resting heart rate of 72 bpm.

The study took place at Klick Inc., which is a technology, media, and research company in the healthcare sector based in Toronto, Canada. All of the participants were employees of Klick Inc. The study was performed in accordance with relevant guidelines and regulations, and all participants signed informed consent. The study received full ethics approval from Advarra IRB Services (www.advarra.com/services/irb-services/).

### Apparatus

The FreeStyle Libre flash glucose monitoring system (Abbott Diabetes Care) was used to measure real-time, continuous interstitial glucose levels with a minimally invasive 5 mm flexible filament inserted into the posterior upper arm. The sensor works based on the glucose-oxidase process by measuring an electrical current proportional to the concentration of glucose. The FreeStyle Libre is a factory calibrated device, designed not to require finger prick tests during use. Previous research has shown the FreeStyle Libre to have consistent accuracy and reliability throughout the 14 days with a mean absolute relative difference of 11.4% compared with capillary blood glucose, and is not significantly influenced by age, sex, body weight, BMI, or time of use (day vs. night)^25–27^.

The device contains a sensor which is attached to the posterior region of the upper arm with an adhesive patch, and a handheld reader device which downloads data from the sensor via near-field communication. Interstitial glucose concentrations (in mmol/L) are captured by the sensor every 15 min and/or when users scan the sensor using the handheld device. The handheld device requires users to scan the sensor at least every 8 h, otherwise previous data are overwritten by the sensor. The system has a lifespan that restricts sensor wear to 14 consecutive days, after which the handheld device will no longer download data from the sensor. In our particular sample, the glucose sensors lasted an average of 13.0 days (due to some cases of malfunction or detachment), with a range of 7–14 days.

### Data collection

At the beginning of the study period, participants completed a self-report demographic survey, and had some physiological variables measured, including height, weight, body mass index (BMI), resting blood pressure, and resting heart rate. Participants were then outfitted with the FreeStyle Libre flash glucose monitor, and instructed on its use. Participants were instructed to scan the sensor with the handheld device at least once every 8 hours to minimize data loss. Missing data were anticipated as participants may have slept over 8 hours, so they were encouraged to scan the device before going to sleep and immediately upon waking. Other than using the glucose device, no other intervention was implemented, and participants were not asked to change their lifestyle in any way.

### Reporting Summary

Further information on research design is available in the Nature Research Reporting Summary linked to this article.

## Supplementary information

Supplementary Information

Data Set 1

Data Set 2

Data Set 3

Data Set 4

Data Set 5

Data Set 6

Reporting Summary

## Data Availability

The data that support the findings of this study are available from the corresponding author upon reasonable request.

## Code availability

All code for this project was written in Python, with reference to the Pandas library for parsing raw data from the FreeStyle Libre device, NumPy for array manipulation and SciPy.interpolate and Matplotlib for producing figures^28^. Apart from functions from these libraries, the code was written from scratch. The detailed description (Supplementary Data Set 1), raw data (Supplementary Data Set 2), and Python code (Supplementary Data Set 3) necessary for reproducing Figs 1 and 2 are available as supplementary material (Supplementary Data Sets 4, 5, 6).
