# Supplementary material for: Homeostasis as a proportional–integral control system
Source: NPJ Digit Med. 2020 May 22;3:77. doi: 10.1038/s41746-020-0283-x (PMC7244502; doi:10.1038/s41746-020-0283-x)
Supplement: Supplementary file 1 — Supplementary Information [file 41746_2020_283_MOESM1_ESM.pdf]

I. SUPPLEMENTARY METHODS

The parameters A_1, A_2 and λ in control model (1), as well as the amplitude and timing of the input peak $F(t)$ in the bood glucose model (2), are specific to each representative peak, extracted from a subject's sequence of measurements. We find the least-squares fit by a simple gradient descent algorithm that relies on the simulation of $u(t)$ and $e(t)$ over a time interval as long as the representative peak.

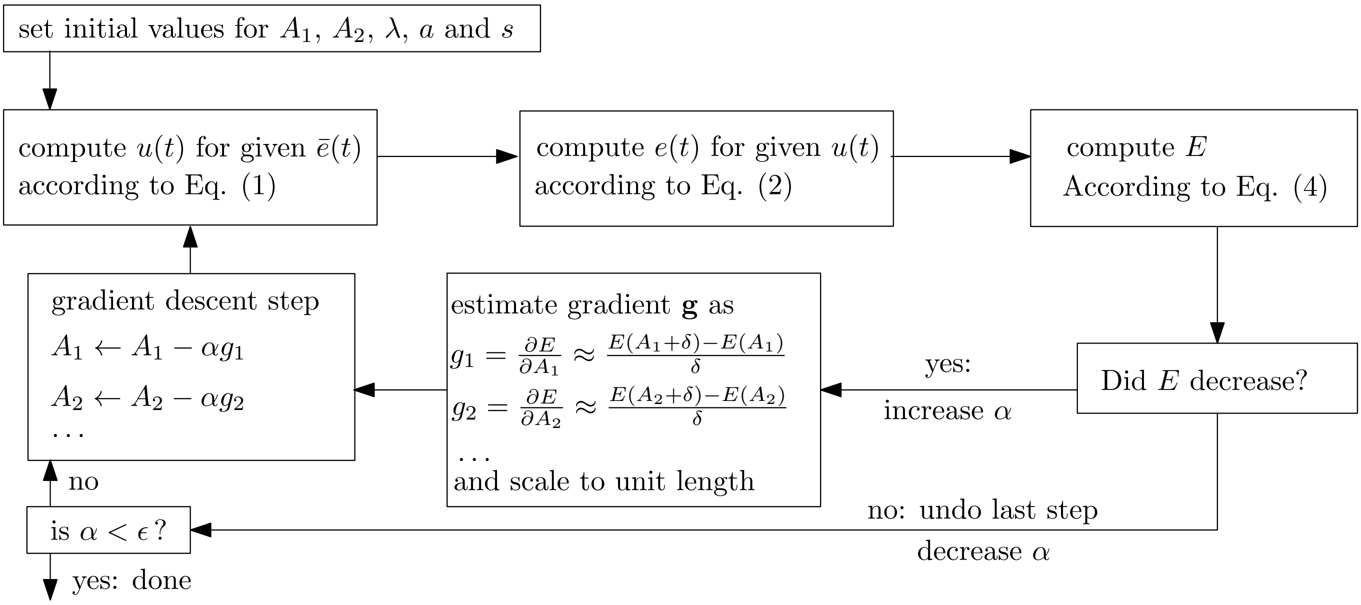

SUPPLEMENTARY FIGURE 1: Schematic representation of the parameter fitting procedure. Starting at the top left, we set initial values for the tunable parameters and set the initial step size to $\alpha = 1$. We then compute the model output $e(t)$ and its difference from the representative peak $\bar{e}$. The gradient of the function E, which measures the mismatch, is approximated by finite differencing with a small constant δ making a 1% variation of the parameter. A gradient descent step of size α is then taken. If E increases from one iteration to the next, the step is rejected and α is decreased. When α is smaller than a pre-set threshold $\epsilon < 10^{-12}$, the algorithm has converged.

The gradient descent algorithm is illustrated in Supplementary Figure 1. For given values of A_1, A_2 and λ, we compute a time series of the control variable $u(t)$ from $\bar{e}(t)$ according to Eq. (1). Next, we obtain the model output $e(t)$ from the control variable and the input function $F(t)$ according to Eq. (2). We assume the input function to take the form of a Gaussian peak with amplitude a, standard deviation Δ and the maximum at $t = s$. While the shape of the input peak does not strongly influence the fitting procedure, its amplitude and the shift of its peak with respect to that of the representative data $\bar{e}$ do. Since we have no a-priori estimates for these quantities, we add them to the list of parameters to be optimized. In addition, the initial value $e(0)$ can be added to this list, but the results remain qualitatively the same if we fix $e(0) = \bar{e}(0)$ for each subject.

From the representative data and the model data, we then compute E according to Eq. (4). Next, we approximate the gradient $\boldsymbol{g}$ of E with respect to the tunable parameters A_1, A_2, λ, a and s by a simple finite difference approximation and scale it to unit length. We then take a gradient descent step in the direction $-\mathbf{g}$ of size α. The step size α is reduced if E does not decrease from one iteration to the next, and increased if it does. The optimal parameter values are found when α decreases below some pre-set threshold.

All code for this project was written in Python, with reference to the Pandas library for parsing raw data from the FreeStyle Libre device, NumPy for array manipulation and SciPy.interpolate and Matplotlib for producing Figure [1]. Apart from functions from these libraries, the code was written from scratch.

SUPPLEMENTARY REFERENCES

[1] See https://pandas.pydata.org/ for Pandas, https://numpy.org/ for NumPy, https://www.scipy.org/ for SciPy and https://matplotlib.org/ for Matplotlib.

II. SUPPLEMENTARY TABLE 1

Sensor code	# Days sensor lasted	Gender	Age	Ethnicity	BMI	Systolic blood pressure (mm Hg)	Diastolic blood pressure (mm Hg)	Resting heart rate (bpm)
0M00081XT20	14	Male	19	Caucasian	21.2	128	75	52
0M00094U0MW	10	Female	26	Asian	23.2	112	73	83
0M000821E1W	11	Male	34	Asian	24.4	111	63	58
0M000981Z38	14	Male	33	Caucasian	22.8	135	71	67
0M0008Y99WR	14	Male	36	Caucasian	29.0	100	72	65
0M0009831M8	14	Male	37	Asian	25.1	139	91	87
0M0008XNLJH	12	Male	37	Caucasian	20.6	104	59	61
0M0008YC85H	10	Female	27	Caucasian	26.2	108	72	59
0M00094TFJR	10	Male	43	Caucasian	23.5	113	71	64
0M00098MTZM	9	Female	26	Caucasian	24.0	125	71	82
0M0008CA77R	14	Female	26	Asian	18.8	94	75	68
0M00098VVGM	14	Female	32	Asian	17.9	103	70	74
0M0008XNLL4	14	Female	42	Caucasian	25.3	114	71	70
0M0007V9G14	13	Female	38	Caucasian	32.2	125	76	73
0M0008YC848	14	Female	29	Caucasian	24.2	121	67	70
0M0008YC83D	7	Male	33	Hispanic	36.9	146	88	67
0M0008CA8Z4	14	Female	34	Caucasian	25.5	108	64	80
0M0008D1F78	12	Male	26	Caucasian and African American	31.5	134	83	62
0M00082GJLD	11	Male	50	Caucasian	27.0	121	83	82
0M00081XT1M	14	Female	22	Caucasian	19.1	111	76	66
0M00098UZDM	14	Male	27	Asian	24.3	106	61	70
0M00082GJMW	14	Male	27	Asian	23.7	123	75	93
0M0008CA728	14	Female	24	Asian	21.1	105	73	65
0M0009TK7CH	10	Female	37	Caucasian	42.4	128	87	72
0M0009TZ6DR	14	Male	33	Caucasian	40.2	163	98	88
0M0008X9MZ4	14	Male	33	Caucasian	35.7	154	91	57
0M0009U0Z4R	14	Male	32	Asian	24.4	110	71	57
0M0009TQA38	14	Female	30	Asian	20.0	108	71	76
0M00048323W	14	Male	27	Caucasian	22.0	127	74	71
0M000829JRM	14	Male	32	Caucasian	24.5	123	93	75
0M0009TAZ8H	14	Male	50	Caucasian	22.5	125	74	66
0M006161C84	14	Male	34	Caucasian	26.5	104	67	77
0M006161T3R	14	Female	37	African American	32.8	120	62	69
0M006161C6M	14	Male	34	Caucasian	25.5	137	72	89
0M0061EKZQM	14	Female	28	Asian	29.1	120	79	90
0M0060ALZX0	14	Female	32	Asian	21.4	120	81	70
0M006161AX8	14	Female	40	Asian	27.6	108	79	81
0M0060PTJHM	14	Female	28	Caucasian	20.8	113	64	71
0M006161T50	14	Female	23	Asian	22	111	60	76
0M006161AVH	14	Male	37	Asian	30.3	135	81	71
0M00615YK4W	14	Female	32	Caucasian	21.1	125	76	66

III. SUPPLEMENTARY DATA SET DESCRIPTIONS

Supplementary Data Set 1: README file about the detailed descriptions of the Supplementary Data Set.

Supplementary Data Set 2: Contains the Python function `selSubject(nr)`. Input is the subject number, which corresponds to the line number in the table of subjects in the Supplementary Information (see above). Output consists of the set point glucose level, the representative peak, the model output, the optimized parameters A_1 and A_2, the standard deviation of the raw blood glucose data and the maximum of the control variable produced by the model. Note, that subject number 0 is empty and can be used to input test data.

Supplementary Data Set 3: Contains a script to read any number of subject data and visualize them. To select subject, set the array "subjects", e.g., `subjects = [1, 16, 36]` to select subjects 1, 16 and 36 or `subjects = [5:24]` to select subjects 5 through 23. For each subject, a time series will be plotted of the representative peak and the model output. In addition a (partial) scatter plot like Fig. 2 from the table is produced.

Supplementary Data Set 4: Raw data file for Inlay A of Figure 2.

Supplementary Data Set 5: Raw data file for Inlay B of Figure 2.

Supplementary Data Set 6: Raw data file for Inlay C of Figure 2.
